# Aligning chemical structure diagrams with local search

**DOI:** 10.1186/1758-2946-4-S1-O4

**Published:** 2012-05-01

**Authors:** Matthias Hilbig, Matthias Rarey

**Affiliations:** 1 Zentrum für Bioinformatik, Universität Hamburg, 20146, Germany

## 

Chemists working in biomolecular application projects are usually looking at many related molecules (e.g. results of a virtual screening run, lead series development or library design). For a convenient visual analysis of this data it is essential that differences between molecules are easily detectable. This can be quite difficult if structural similarities are not taken into account while creating molecule structure diagrams. We present a method for generating globally aligned structure diagrams for two molecules following IUPAC standards [1]. Using a set of three coordinate transform operations (ring system flipping, chain flipping and substituent exchange) all correct and overlap-free layouts can be enumerated. If the number of possible layouts is too large, a heuristic is used to iterate through a smaller subspace. Subsequently all candidate layouts are scored with several different terms describing the quality of the layout (number of collisions, stretching of chains...) as well as the relationship between molecules (similarity to a template) and the one with the highest score is chosen. Scoring functions and similarity measures are easily interchangeable and the whole process is fast enough for interactive use. The whole alignment process is verified by calculating the RMSD between aligned and nearest template coordinates. For validation, the new method is applied to many clusters of related molecules from the PubChem compound library. In summary, we have developed a novel SDG algorithm which is of great help for the daily tasks of a modeller by drawing small, related molecules consistently.

**Figure 1:**
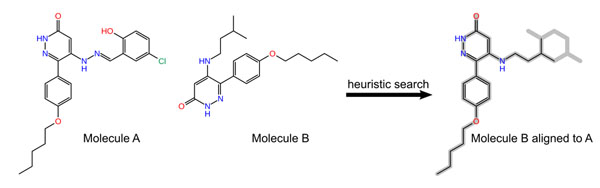


## References

[B1] Brecher J Graphical representation standards for chemical structure diagrams (IUPAC Recommendations 2008) Pure Appl Chem 2008 80 277 410 10.1351/pac200880020277

[B2] Fricker PC Gastreich M Rarey M Automated Drawing of Structural Molecular Formulas under Constraints J Chem Inf Comput Sci 2004 44 1065 1078 10.1021/ci049958u 15154775

[B3] Boissonnat J Cazals F Flötotto J 2D - structure drawings of similar molecules Graph Drawing 2001 1984 89 93

[B4] Clark AM 2D depiction of fragment hierarchies J Chem Inf Model 2010 50 37 46 10.1021/ci900350h 20038186 PMC2810838

